# Baseline IL-6 is a biomarker for unfavorable tuberculosis treatment outcomes: a multi-site discovery and validation study

**DOI:** 10.1183/13993003.00905-2021

**Published:** 2022-04-21

**Authors:** Akshay N. Gupte, Pavan Kumar, Mariana Araújo-Pereira, Vandana Kulkarni, Mandar Paradkar, Neeta Pradhan, Pradeep Menon, Padmapriya Darasini, Luke-Elizabeth Hanna, Shri Vijay Bala Yogendra Shivakumar, Neesha Rockwood, Elsa Du Bruyn, Rajesh Karyakarte, Sanjay Gaikwad, Robert Bollinger, Jonathan Golub, Nikhil Gupte, Vijay Viswanathan, Robert J. Wilkinson, Vidya Mave, Subash Babu, Hardy Kornfeld, Bruno B. Andrade, Amita Gupta

**Affiliations:** 1 Division of Infectious Diseases, Johns Hopkins University School of Medicine, Baltimore, USA; 2 Center for Clinical Global Health Education, Johns Hopkins University School of Medicine, Baltimore, USA; 3 National Institute for Research in Tuberculosis, Chennai, India; 4 Instituto Gonçalo Moniz, Fundação Oswaldo Cruz (FIOCRUZ), Salvador, Brazil; 5 Multinational Organization Network Sponsoring Translational and Epidemiological Research, Salvador, Brazil; 6 Faculdade de Medicina, Universidade Federal da Bahia, Salvador, Brazil; 7 Byramjee-Jeejeebhoy Government Medical College-Johns Hopkins University Clinical Research Site, Pune, India; 8 Johns Hopkins India Private Limited, Pune, India; 9 Wellcome Centre for Infectious Disease Research in Africa, Institute of Infectious Disease and Molecular Medicine, University of Cape Town, Observatory, South Africa; 10 Department of Microbiology, Faculty of Medicine, University of Colombo, Colombo 8, Sri Lanka; 11 Department of Infectious Diseases, Imperial College London, W12 ONN, United Kingdom; 12 Department of Infectious Diseases, University of Cape Town, Observatory, South Africa; 13 Department of Microbiology, Byramjee-Jeejeebhoy Government Medical College, Pune, India; 14 Department of Pulmonary Medicine, Byramjee-Jeejeebhoy Government Medical College, Pune, India; 15 Center for Tuberculosis Research, Johns Hopkins University, Baltimore, USA; 16 Professor M. Viswanathan Diabetes Research Center, Chennai, India; 17 The Francis Crick Institute, 1 Midland Rd, London NW1 1AT; 18 National Institutes of Health – National Institute for Research in Tuberculosis – International Center for Excellence in Research, Chennai, India; 19 Division of Pulmonary Medicine, University of Massachusetts Medical School, Worcester, USA

## Abstract

**Background:**

Biomarkers of unfavorable tuberculosis treatment outcomes are needed to accelerate new drug and regimen development. Whether plasma cytokine levels can predict unfavorable tuberculosis treatment outcomes is unclear.

**Methods:**

We identified and internally validated the association between 20 a-priori selected plasma inflammatory markers and unfavorable treatment outcomes of failure, recurrence and all-cause mortality among adults with drug-sensitive pulmonary tuberculosis in India. We externally validated these findings in two independent cohorts of predominantly diabetic and HIV coinfected tuberculosis patients in India and South Africa, respectively.

**Results:**

Pre-treatment IFN-γ, IL-13 and IL-6 were associated with treatment failure in the discovery analysis. Internal validation confirmed higher pre-treatment IL-6 concentrations among failure cases compared to controls. External validation among predominantly diabetic tuberculosis patients found an association between pre-treatment IL-6 concentrations and subsequent recurrence and death. Similarly, external validation among predominantly HIV coinfected tuberculosis patients found an association between pre-treatment IL-6 concentrations and subsequent treatment failure and death. In a pooled analysis of 363 tuberculosis cases from the Indian and South African validation cohorts, high pre-treatment IL-6 concentrations were associated with higher risk of failure (adjusted odds ratio [aOR]=2.16, 95%CI 1.08-4.33, p=0.02), recurrence (aOR=5.36, 95%CI 2.48-11.57, p<0.001) and death (aOR=4.62, 95%CI 1.95-10.95, p<0.001). Adding baseline IL-6 to a risk-prediction model comprising of low BMI, high smear grade and cavitation improved model performance by 15 percent (C-statistic of 0.66 versus 0.76, p=0.02).

**Conclusion:**

Pre-treatment IL-6 is a biomarker for unfavorable tuberculosis treatment outcomes. Future studies should identify optimal IL-6 concentrations for point-of-care risk prediction.

## Introduction

Tuberculosis is the leading infectious cause of death worldwide with over 10 million new cases and 1.5 million deaths annually(1). Drug-sensitive cases require six months of multi-drug therapy for durable cure. Although effective in the majority of cases, implementing this relatively long and complex treatment regimen can increase risk of default and overutilization of health system resources. Developing shorter and simpler tuberculosis treatment regimens is therefore a research priority. Biomarkers have the potential to accelerate new drug and regimen discovery by identifying tuberculosis patients at high risk of unfavorable treatment outcomes who can preferentially be enrolled into early phase clinical trials, or conversely, characterize a subgroup of low-risk patients who may benefit from shorter duration of therapy(2).

Sputum culture conversion at two months of treatment is the most widely used biomarker of treatment response; however, its validity in predicting subsequent clinical outcomes has been questioned in recent treatment shortening trials(3, 4). Blood based biomarkers have an advantage as they overcome the difficulty of reliably obtaining high quality sputum and can be translated into point-of-care tests. A recent study by Kumar et al identified an association between a combination of six chemokines and unfavorable tuberculosis treatment outcomes, suggesting a role of plasma inflammatory markers as predictors of unfavorable treatment outcomes(5). However, translating such a “signature” comprising of multiple and often correlated immune markers, each with their different concentration threshold for optimal risk-prediction, to a point-of-care test is challenging. Whether a parsimonious selection of inflammatory markers can predict unfavorable treatment outcomes in tuberculosis patients is unclear.

We have previously identified plasma cytokines associated with lung injury in pulmonary tuberculosis cases in India(6). We now report on a multi-site discovery-validation analysis in India and South Africa to identify plasma cytokines associated with treatment failure, recurrence and death.

## Methods

### Study design and population

We conducted a prospective cohort study to discover plasma cytokines associated with unfavorable treatment outcomes among adult drug-sensitive pulmonary tuberculosis patients enrolled in the Cohort for Tuberculosis Research by the Indo-US Medical Partnership (CTRIUMPH) study in Pune, India(7). We internally validated these results among CTRIUMPH participants not previously included in the discovery cohort by nesting a 1:1 age- and sex-matched case-control analysis among adult drug-sensitive pulmonary tuberculosis patients who failed treatment (cases) and those who were cured (controls). We subsequently conducted external validation analyses in two independent cohorts from India and South Africa. The Indian external validation cohort comprised of predominantly diabetic drug-sensitive pulmonary tuberculosis patients from the Effect of Diabetes on Tuberculosis Severity (EDOTS) study in Chennai, India(8). In this cohort, we nested a 1:2 age-, sex- and body-mass-index (BMI)-matched case-control analysis among adult drug-sensitive pulmonary tuberculosis patients who experienced a composite unfavorable treatment outcome (cases) of failure, recurrence or death, and those with recurrence free cure over 18 months of follow-up (controls). The South African external validation cohort comprised of predominantly HIV coinfected drug-sensitive pulmonary tuberculosis patients from Khayelitsha, South Africa(9, 10). In this cohort, we conducted an unmatched case-control analysis among adult drug-sensitive pulmonary tuberculosis patients who experienced a composite unfavorable treatment outcome (cases) of failure, recurrence or death, and those with recurrence free cure over 18 months of follow-up (controls). Finally, we pooled data from the Indian and South African cohorts for a combined validation analysis. Detailed descriptions of the discovery, internal validation, Indian external validation and South African external validation cohorts are provided in Supplementary Appendix 1. In addition to different study populations, we used different laboratories for measuring cytokine concentrations for the internal and external validation studies to assess generalizability of our findings (Supplementary Table-1). This study was approved by the Ethics Committees at the participating study sites in India and South Africa.

### Cytokine measurement

For the discovery analysis, plasma samples collected at treatment initiation, 2 months and 6 months underwent cytokine testing, in duplicates, using multiplex ELISA on Luminex assay (Bio-Rad, USA) at the National Institutes of Health (NIH) – National Institute for Research in Tuberculosis (NIRT) – International Center for Excellence in Research (ICER) laboratory in Chennai, India. Cytokines analyzed were selected a-priori for their role in the host inflammatory response to *Mycobacterium tuberculosis* (interferon gamma [INF-γ], tumor necrosis factor alpha [TNF-α], interleukin [IL]-1β, IL-4, IL-6, IL-10, IP-10, IL-12, IL-13 and IL-17)(11), lung pathology (matrix metalloproteinases [MMP]-1, MMP-3, MMP-7, tissue inhibitor of metalloprotease [TIMP]-1, TIMP-2, TIMP-3, TIMP-4)(12), and fibrous remodeling (transforming growth factor beta [TGFβ]-1, TGFβ-2, TGFβ-3)(13). IL-6, IL-13 and IFN-γ measured at treatment initiation were statistically significantly associated with treatment failure in the discovery analysis and were therefore selected for internal validation. For the internal validation analysis, plasma samples collected at treatment initiation underwent cytokine testing, in duplicates, using multiplex ELISA by Luminex assay (Bio-Rad, USA) at the Byramjee-Jeejeebhoy Government Medical College (BJGMC) laboratory in Pune, India. IL-6 measured at treatment initiation was statistically significantly associated with treatment failure during internal validation and was therefore selected for independent external validation. For the Indian external validation cohort, plasma samples collected at treatment initiation underwent IL-6 testing by Luminex assay (R&D Systems, USA) at the NIH-NIRT-ICER laboratory in Chennai, India. For the South African external validation cohort, plasma samples collected at tuberculosis treatment initiation underwent IL-6 testing by Luminex assay (MilliporeSigma, USA) at the Wellcome Centre for Infectious Disease Research, South Africa. The lower limit of IL-6 detection was 0.31 pg/mL and a nonlinear curve fit model was used to estimate IL-6 values less than the lower standard values.

### Outcomes

Unfavorable tuberculosis treatment outcomes included failure, recurrence and death. Treatment failure was defined as culture confirmation of *M tuberculosis* during the last two months of treatment. Recurrence was defined in a tuberculosis patient who did not fail treatment but was subsequently found to have culture positive tuberculosis during 18 months of post-treatment follow-up. Death included all-cause mortality.

### Statistical analysis

#### Discovery

We compared cytokine expression at treatment initiation, 2 months and 6 months between tuberculosis cases with and without an unfavorable treatment outcome using one-way hierarchical cluster analysis by Ward’s method with 100x bootstrap. Median concentrations of cytokines, stratified by treatment outcomes and duration, were used to describe the overall change in cytokine expression in response to tuberculosis treatment. Cytokine concentrations were log_10_-transformed, or z-score normalized for analysis. Data were compared using the Mann-Whitney U test and P-values were adjusted for multiple comparisons using the Holm-Bonferroni method(15).

#### Internal and external validation

We compared log_10_-transformed cytokine concentrations between participants with and without an unfavorable treatment outcome using the Wilcoxon sign-rank test. Receiver Operator Characteristic Curve (ROC) analysis was used to assess the ability of individual cytokines to discriminate between participants with and without unfavorable treatment outcomes. Random-effects linear regression, with matched case-control pairs as random effects, was used to compare cytokine concentrations between participants with and without unfavorable treatment outcomes. Multivariable analyses were adjusted for tuberculosis disease severity assessed by BMI, chest radiography (CXR) score including cavitation, and sputum smear grade. We further adjusted for pre-treatment illness duration to account for differences in the timing of patient presentation relative to disease onset and its possible impact on cytokine concentrations, glycated hemoglobin (HbA1c) among participants with diabetes and, CD4 cell counts and receipt of antiretroviral therapy (ART) among HIV coinfected participants.

#### Pooled validation

We pooled data from the internal and external validation cohorts to measure the association between baseline IL-6 concentrations and unfavorable tuberculosis treatment outcomes. Since each of the three validation cohorts utilized different laboratories, ELISA kits and testing protocols, there was substantial variability in picogram/milliliter (pg/mL) IL-6 quantification across the study sites. To account for this variability, we used z-scores and percentiles to standardize IL-6 concentrations across the three validation cohorts. For each validation cohort, we calculated a z-score for pg/mL IL-6 concentrations using the formula *z = (observed concentration – mean concentration) /standard deviation*. This standardized z-score was used for ROC analysis in the pooled validation cohort. Similarly, we classified “high” IL-6 concentrations as having pg/mL concentrations in the highest quartile for a given study. “Low” IL-6 concentrations were defined as having pg/mL concentrations in the lower three quartiles for a given study. To account for clustering by study location and case-control matching, we used random-effects logistic regression with bootstrap confidence intervals, to measure the association between “high” baseline IL-6 concentrations and unfavorable tuberculosis treatment outcomes of failure, recurrence or death. Multivariable analyses adjusted for age, sex, BMI, smear grade, CXR score including cavitation, pre-treatment illness duration, HIV and diabetes. Furthermore, cavitary disease, low BMI and higher smear grade have previously been shown to predict unfavorable tuberculosis treatment outcomes(16). Therefore, we calculated the incremental gain in discriminatory ability, measured by the C-statistic, by adding baseline IL-6 to a risk-prediction model comprising of cavitation, BMI and smear grade.

## Results

**Figure 1:**
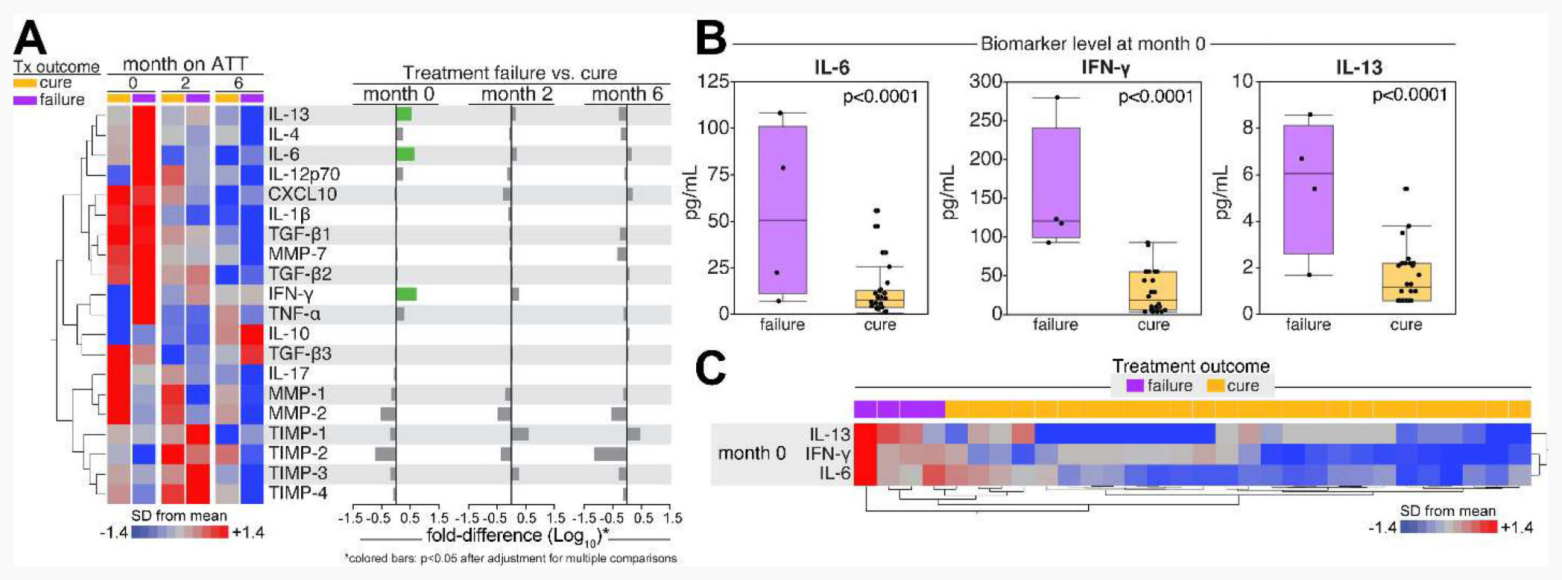
Cytokines associated with treatment failure in the discovery cohort. **Panel A:** Differences in cytokine concentrations (z-score standardized and log_10_-transformed) between participants who failed treatment and those who were cured, stratified by duration of treatment. **Panel B:** Differences in absolute cytokine concentrations (pg/mL) at enrollment between participants who failed treatment and those who were cured. **Panel C:** A two-way unsupervised hierarchical cluster analysis using concentrations of IL-6, IFN-γ and IL-13 measured at enrollment to identify a unique combined profile of biomarker protein expression that could distinguish participants based on treatment outcome.

**Figure 2:**
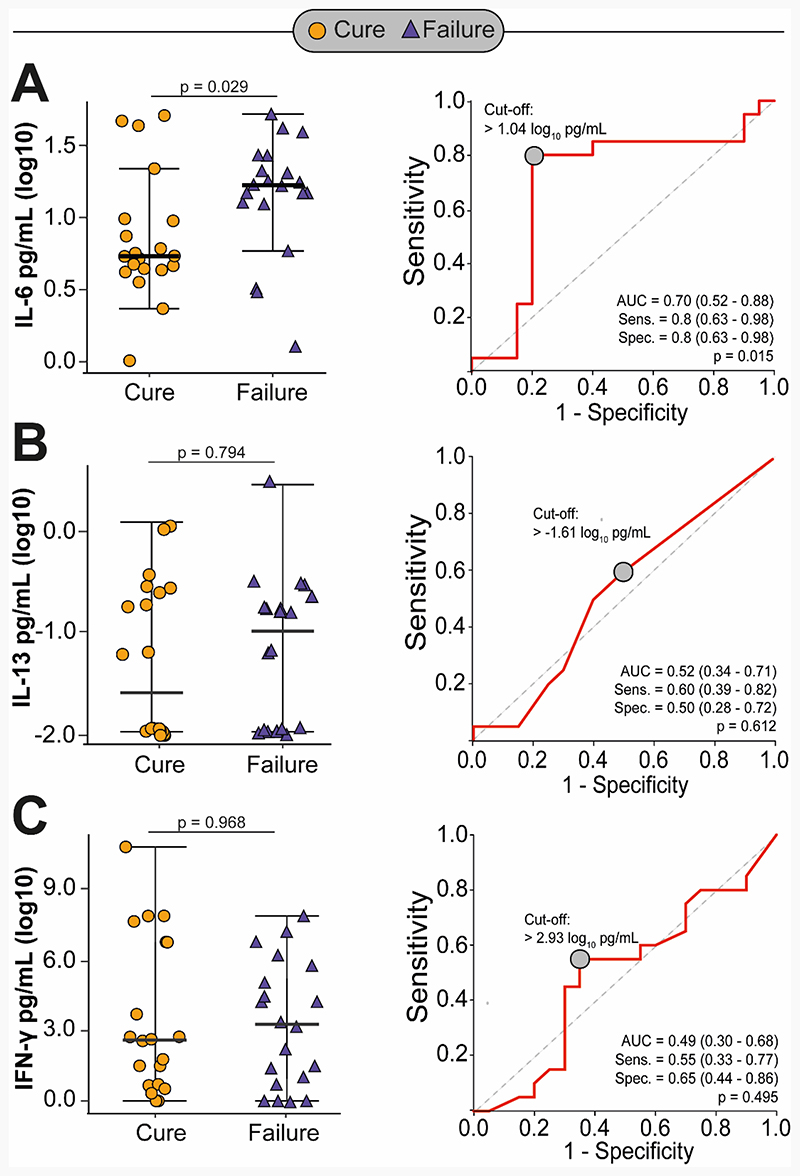
Difference in IL-6 (Panel A), IL-13 (Panel B) and IFN-γ (Panel C) concentrations between tuberculosis treatment failures and cures, and associated area under the curve (AUC) for failure-cure classification in the internal validation cohort.

Baseline characteristics of the discovery sub-cohort did not differ significantly from the full cohort and are summarized in Supplementary Table-2. Of the 30 participants selected in the discovery sub-cohort, 4 (13%) failed treatment, and none had recurrence or died. Participants who failed treatment had significantly higher baseline concentrations of IL-6, INF-γ and IL-13 relative to those without failure (p<0.001 for all); however, we did not find statistically significant differences in cytokine levels measured at 2 and 6 months of treatment (Figure-1). The internal validation cohort comprised of 20 participants with culture confirmed treatment failure and 20 age- and sex-matched participants with culture confirmed cure. Overall, 3 (8%) participants were HIV coinfected and 3 (8%) had diabetes. Except for lower median BMI (16.0 vs 17.4 kg/m^2^, p=0.02) and longer pre-treatment illness duration (35 vs 30 days, p=0.03), cases were comparable to controls with respect to their baseline characteristics (Supplementary Table-3). After adjusting for markers of disease severity and pre-treatment illness duration, cases had significantly higher IL-6 concentrations at baseline relative to controls (0.26 log-higher concentration, 95%CI 0.01 to 0.52, p=0.04) with an area under the curve (AUC) of 0.70 (95%CI 0.52-0.88). We did not find differences in INF-γ and IL-13 concentrations by treatment outcomes (Table-1 and Figure-2).

**Figure 3:**
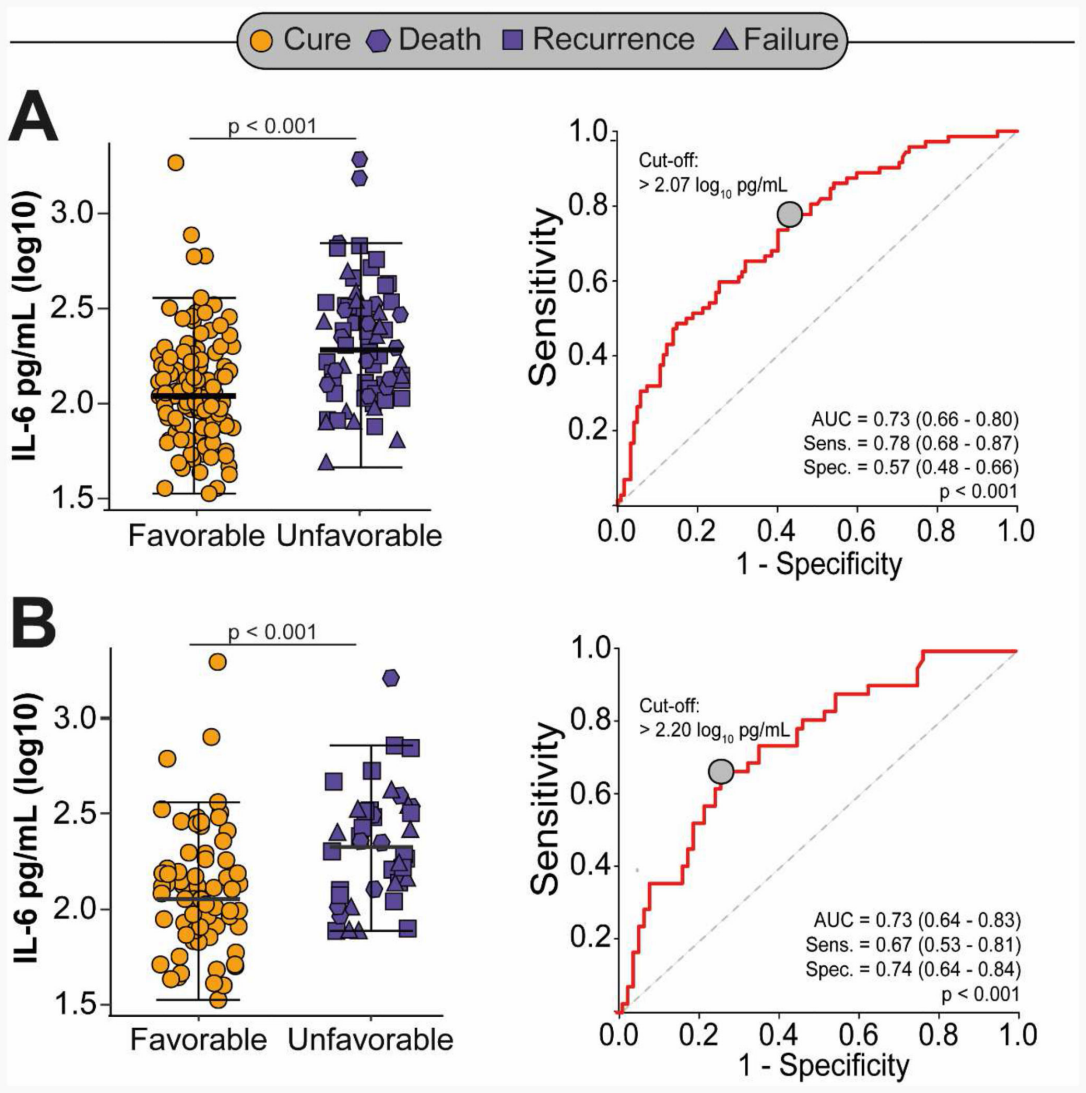
Difference in IL-6 concentrations between tuberculosis patients with and without unfavorable treatment outcomes, and associated area under the curve (AUC) among all participants (Panel A) and restricted to patients with diabetes only (Panel B) in the Indian external validation cohort.

The Indian external validation cohort was comprised of 72 cases with an unfavorable treatment outcome and 122 age-, sex- and BMI-matched controls with recurrence free cure. Overall, 115 (59%) participants had diabetes and HIV was an exclusion criterion. Cases were comparable to controls with respect to their baseline characteristics, except for a higher proportion of ever-smokers (Supplementary Table-4). Overall, 18 (9%) participants failed treatment, 35 (18%) had recurrence, and 19 (10%) died. Cases with an unfavorable treatment outcome had significantly higher concentrations of IL-6 at baseline relative to controls (0.26 log-higher concentration, 95%CI 0.17 to 0.36, p<0.001), after adjusting for markers of disease severity, pre-treatment illness duration, smoking status and diabetes. Results were similar when restricted to tuberculosis patients with diabetes and adjusting for HbA1c levels; cases experiencing recurrence or death had higher baseline IL-6 concentrations compared to controls (Table-2 and Figure-3). IL-6 could effectively discriminate tuberculosis patients with and without unfavorable treatment outcomes with an AUC of 0.73 (95%CI 0.66-0.80) for all participants and 0.73 (95%CI 0.64-0.83) when restricted to participants with diabetes (Figure-3).

**Figure 4:**
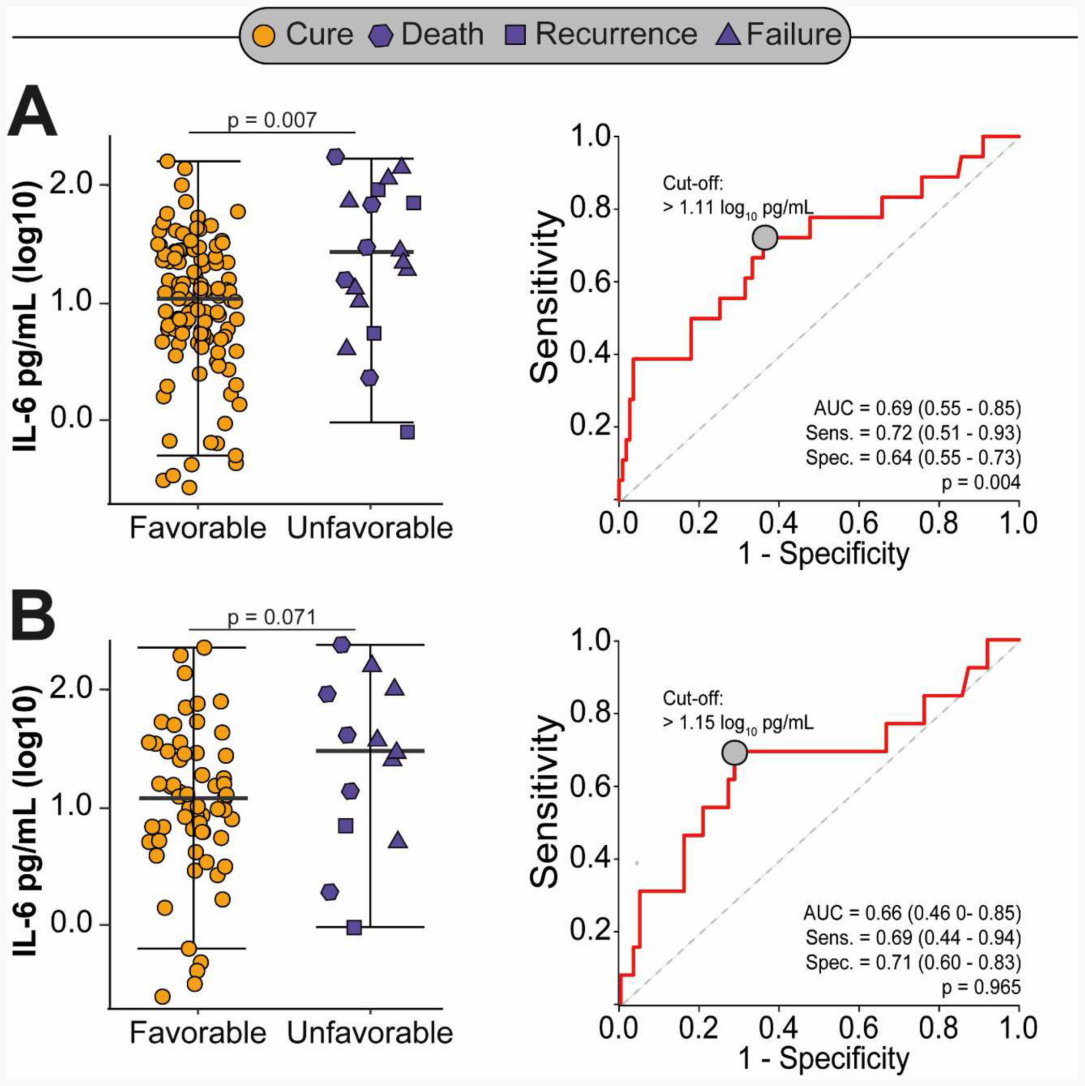
Difference in IL-6 concentrations between tuberculosis patients with and without unfavorable treatment outcomes, and associated area under the curve (AUC) among all participants (Panel A) and restricted to HIV coinfected patients only (Panel B) in the South African external validation cohort.

Of the 129 participants enrolled in the South African external validation cohort, 76 (59%) had HIV, 29 (38%) were receiving antiretroviral therapy (ART) and the median (IQR) CD4 count was 192 (66-366) cells/mm^3^ (Supplementary Table-5). Overall, 18 (14%) participants experienced an unfavorable treatment outcome; 9 (7%) failed treatment, 4 (3%) had recurrence and 5 (4%) died. After adjusting for age, sex, HIV status, ART and markers of disease severity, participants who experienced an unfavorable treatment outcome had significantly higher concentrations of IL-6 at baseline compared to those with recurrence free cure (0.38 log-higher concentration, 95%CI 0.13 to 0.63, p=0.003). However, we found marginally significant associations between higher baseline IL-6 concentrations and unfavorable tuberculosis treatment outcomes when restricted to HIV coinfected participants (Table-3). IL-6 could discriminate tuberculosis patients with and without unfavorable treatment outcomes with an AUC of 0.69 (95%CI 0.55-0.85) for all participants and 0.66 (95%CI 0.42-0.85) when restricted to HIV coinfected participants (Figure-4).

The pooled validation from Indian and South African cohorts comprised of 363 participants experiencing 110 unfavorable outcomes: 47 (13%) had failure, 39 (11%) had recurrence and 24 (6%) died. High IL-6 concentration at baseline was present in 91 (25%) participants and was associated with 3-fold higher odds of any subsequent unfavorable treatment outcome (95%CI 2.03-5.89, p<0.001) after adjusting for age, sex, BMI, CXR score including cavitation, smear grade, pre-treatment illness duration, HIV, diabetes and correlations within each contributing study (Table-4). Similarly, high baseline IL-6 concentrations were significantly associated with subsequent failure (adjusted odds ratio [aOR]=2.22, 95%CI 1.04-4.75, p=0.03), recurrence (aOR=5.92, 95%CI 2.52-13.90, p<0.001) and death (aOR=5.53, 95%CI 2.08-14.68, p<0.001) in adjusted analyses.

Low BMI, high smear grade (≥ 2+) and cavitation on CXR were poor baseline predictors of unfavorable tuberculosis treatment outcomes in our cohort. A risk-prediction model comprising of these variables had a C-statistic (AUC) of 0.66 (95%CI 0.56-0.77) for discriminating between tuberculosis patients with and without unfavorable treatment outcomes. However, baseline IL-6 could discriminate tuberculosis patients with and without unfavorable treatment outcomes with an AUC of 0.71 (95%CI 0.65-0.77); a z-score cut-off of -0.32 identified by the Youden’s method had a sensitivity of 76%, specificity of 60%, and a negative predictive value of 85% for ruling out subsequent unfavorable tuberculosis treatment outcomes. Furthermore, the addition of baseline IL-6 improved performance of the prediction model comprising of low BMI, high smear grade and cavitation by 15 percent (C-statistic=0.76, 95%CI 0.67-0.85, p=0.02) (Supplementary Table-6).

## Discussion

Our discovery-validation analyses among geographically and epidemiologically diverse populations of pulmonary tuberculosis cases found an association between higher baseline IL-6 concentrations in plasma and subsequent treatment failure, recurrence and death. This association was generalizable despite different testing laboratories, comorbidities of diabetes and HIV, and after adjusting for disease severity. Furthermore, the inclusion of baseline IL-6 significantly improved the performance of established risk-prediction models for poor clinical outcomes. Our data support the role of IL-6 as a biomarker for unfavorable tuberculosis treatment outcomes.

IL-6 exhibits a pleotropic immunoregulatory role by promoting neutrophil and macrophage recruitment and survival, inducing the acute phase response in the liver, and facilitating tissue damage by stimulating protease secretion and matrix deposition(17, 18). IL-6 has long been considered a non-specific marker of tuberculosis disease activity and, prior studies have identified a correlation between IL-6 concentrations, bacterial burden and lung pathology(6, 19, 20). However, few studies have reported an association between IL-6 and tuberculosis treatment outcomes. A secondary analysis of an early phase treatment trial reported correlations between tuberculosis disease severity and baseline IL-6 concentrations, and between greater declines in IL-6 concentrations and sputum culture conversion during the first two months of treatment(21). However, this study did not report on final treatment outcomes. Across the three independent cohorts analyzed in our study, we found a consistent association between higher baseline IL-6 concentrations and subsequent unfavorable tuberculosis treatment outcomes. This association remained significant after adjusting for markers of disease severity and suggests an independent predictive role of baseline IL-6 for risk-stratification in tuberculosis patients.

While our study found an association of baseline IL-6 concentrations with recurrent tuberculosis in participants with and without diabetes, we did not find a similar association among HIV coinfected participants. This finding is inconsistent with a recent study by Sivro et al., which reported an association between IL-6 concentrations, measured after completion of tuberculosis treatment, and recurrent disease among ART naïve HIV coinfected patients(22). This inconsistency may be due to differences in the underlying study populations and timing of IL-6 measurement – treatment initiation in our study compared to after treatment completion in the Sivro et al study. However, our sample size for HIV-restricted analysis was limited and further validation in larger cohorts of HIV coinfected tuberculosis patients is needed. Conversely, we found higher baseline IL-6 concentrations among tuberculosis patients with diabetes who had recurrence or died; we did not find similar differences by treatment failure. The precise reasons for this are unclear. The internal validation cohort was specifically designed to examine an outcome of treatment failure, while the external validation cohorts did not perform a-priori sampling on treatment outcomes. A relatively fewer number of treatment failure cases in the external validation cohorts may explain a weaker association of baseline IL-6 with treatment failure. Furthermore, diabetes is characterized by hyperinflammation and higher overall IL-6 concentrations among participants with diabetes at the start of tuberculosis treatment could explain this finding(23–25). Nevertheless, further study is needed explore longitudinal changes in the immunological profile of tuberculosis patients with diabetes who fail treatment.

A consistent finding in our study, regardless of HIV or diabetes comorbidity, was the association between baseline IL-6 with all-cause mortality. The ability of IL-6 to predict mortality has previously been reported in several non-communicable and infectious diseases, and more recently in COVID-19(26–30). While studies among tuberculosis patients with advanced HIV do suggest a role of IL-6 in explaining excess mortality(31, 32), similar associations among HIV uninfected tuberculosis patients have yet to be investigated. Our study addresses this knowledge gap by reporting a consistent and significant association between high baseline IL-6 concentrations and subsequent all-cause mortality in HIV uninfected tuberculosis patients with and without diabetes. Importantly, majority of deaths in our study occurred during tuberculosis treatment and, the association between IL-6 and mortality was independent of baseline disease severity and duration of symptomatic illness prior to testing. These data suggest an independent role of IL-6 mediated immune pathways in mortality during tuberculosis treatment.

An important limitation of our study was the relatively small discovery cohort and a limited selection of cytokines. We selected cytokines a-priori based on their biological plausibility; however, a non-targeted approach may identify additional cytokines predictive of unfavorable outcomes. Further, our randomly selected discovery cohort was exploratory in nature and did not include participants with recurrence or death. Therefore, we are likely underpowered to detect a statistically significant association of a smaller magnitude for selected cytokines. We did not objectively assess treatment adherence. However, we did measure self-reported treatment adherence and all participants reported taking over 95% of their prescribed doses. Finally, each of the validation cohorts used a different laboratory, ELISA kits and testing protocols which led to a high variability in IL-6 quantification. Therefore, we were unable to identify an IL-6 pg/mL concentration cut-off for point-of-care risk-prediction.

Despite these limitations, our study found a significant and generalizable association between high baseline IL-6 concentrations and subsequent unfavorable tuberculosis treatment outcomes in a relatively large and epidemiologically diverse population. Although the overall discriminatory ability of IL-6 in our study was modest, it was nevertheless consistent across the Indian and South African validation cohorts. Identifying concentration thresholds to optimize either sensitivity or specificity could facilitate the use of baseline IL-6 as a rule-out or rule-in test, respectively, for risk-stratification at treatment initiation. Such an approach may be important given the poor performance of low BMI, high smear grade and cavitary disease, clinical characteristics that have recently been shown to identify hard-to-treat phenotypes of tuberculosis patients in clinical trial settings(16), in predicting unfavorable treatment outcomes in programmatic settings as seen in this study. For instance, the inclusion of baseline IL-6 in a risk-prediction model comprising of these clinical variables significantly improved performance of the prediction model by nearly 15 percent in our study, supporting a strategy of combining baseline IL-6 with established clinical predictors of unfavorable outcomes for risk-stratification in tuberculosis. Furthermore, in contrast to prior studies which identified predictive signatures comprising of multiple immune markers, our data identified a single cytokine predictive of unfavorable tuberculosis treatment outcomes, potentially simplifying its translation to a point-of-care assay. Future studies should focus on standardizing IL-6 measurements across laboratories for point-of-care translation, and identify optimal predictive thresholds of IL-6 concentrations for risk-stratification.

## Supplementary Material

Supplementary Appendix

## Figures and Tables

**Table 1:** Difference in cytokine concentrations between tuberculosis patients who failed treatment (cases) and those who were cured (controls) in the internal validation cohort.

Cytokines	Log_10_ difference in baseline cytokine levels comparing cases to controls			
	*Unadjusted difference (95% CI)*	*p-value*	*Adjusted difference (95% CI)*	*p-value*
**IL-6**	0.29 (0.06 to 0.52)	0.01	0.26 (0.01 to 0.52)	0.04
**IFN-γ**	-0.12 (-0.68 to 0.42)	0.65	-0.38 (-0.98 to 0.21)	0.21
**IL-13**	0.09 (-0.20 to 0.39)	0.52	0.13 (-0.22 to 0.50)	0.45

Adjusted analysis account for BMI, CXR score including cavitation, smear grade and pre-treatment illness duration. Age and sex are adjusted by study design. CI – confidence interval.

**Table 2:** Difference in IL-6 concentrations between with tuberculosis patients with and without unfavorable treatment outcomes in the Indian external validation cohort.

Treatment outcomes	Log_10_ difference in baseline cytokine levels comparing cases to controls			
	*Unadjusted difference (95%CI)*	*p-value*	*Adjusted difference (95%CI)*	*p-value*
**All participants**				
Composite	0.24 (0.16 to 0.32)	<0.001	0.26 (0.17 to 0.36)	<0.001
Failure	0.10 (-0.02 to 0.24)	0.10	0.16 (-0.02 to 0.36)	0.07
Recurrence	0.26 (0.17 to 0.35)	<0.001	0.27 (0.16 to 0.38)	<0.001
Death	0.32 (0.18 to 0.46)	<0.001	0.32 (0.14 to 0.50)	<0.001
**Diabetes only**				
Composite	0.23 (0.12 to 0.35)	<0.001	0.22 (0.07 to 0.37)	0.01
Failure	0.15 (-0.03 to 0.34)	0.11	0.15 (-0.12 to 0.43)	0.27
Recurrence	0.22 (0.10 to 0.36)	0.001	0.25 (0.05 to 0.44)	0.01
Death	0.30 (0.10 to 0.50)	0.004	0.26 (0.01 to 0.51)	0.04

Adjusted analysis account for CXR score including cavitation, smear grade, ever smoking, diabetes and pre-treatment illness duration. Age, sex and BMI are adjusted by study design. Diabetes-restricted analysis are further adjusted for HbA1c levels. CI – confidence interval.

**Table 3:** Difference in IL-6 concentrations between with tuberculosis patients with and without unfavorable treatment outcomes after adjusting for markers of disease severity in the South African external validation cohort.

Treatment outcomes	Log_10_ difference in baseline cytokine levels comparing participants with unfavorable treatment outcomes to cures			
	*Unadjusted difference (95%CI)*	*p-value*	*Adjusted difference (95%CI)*	*p-value*
**All participants**				
Composite	0.33 (0.06 to 0.60)	0.01	0.38 (0.13 to 0.63)	0.003
Failure	0.41 (0.05 to 0.76)	0.02	0.49 (0.15 to 0.82)	0.004
Recurrence	0.10 (-0.43 to 0.64)	0.70	-0.08 (-0.60 to 0.42)	0.73
Death	0.38 (-0.01 to 0.86)	0.10	0.50 (0.06 to 0.94)	0.02
**HIV coinfected only**				
Composite	0.22 (-0.10 to 0.55)	0.15	0.29 (-0.01 to 0.60)	0.06
Failure	0.39 (-0.04 to 0.83)	0.06	0.46 (0.01 to 0.90)	0.04
Recurrence	-0.64 (-1.39 to 0.10)	0.10	-0.56 (-1.23 to 0.12)	0.10
Death	0.36 (-0.12 to 0.85)	0.13	0.48 (0.05 to 0.92)	0.03

Adjusted analyses account for age, sex, BMI, cavitation on chest X-ray, smear grade and HIV. HIV-restricted analyses are further adjusted for CD4 cell count and ART receipt. CI – confidence interval.

**Table 4:** Association between high concentration of IL-6 at baseline and unfavorable tuberculosis treatment outcomes in the pooled validation analysis.

Treatment outcomes	Odds ratios for unfavorable treatment outcomes			
	*Unadjusted (95%CI)*	*p-value*	*Adjusted (95%CI)*	*p-value*
Composite	3.32 (2.02-5.46)	<0.001	3.45 (2.03-5.89)	<0.001
Failure	2.16 (1.08-4.33)	0.02	2.22 (1.04-4.75)	0.03
Recurrence	5.36 (2.48-11.57)	<0.001	5.92 (2.52-13.90)	<0.001
Death	4.62 (1.95-10.95)	<0.001	5.53 (2.08-14.68)	<0.001

Regression analyses are adjusted for age, sex, BMI, cavitation on CXR, smear grade, HIV and diabetes.

## Take home message

Pre-treatment IL-6 is a biomarker for unfavorable tuberculosis treatment outcomes independent of disease severity and, improves the performance of risk-prediction models comprising of established clinical predictors.
