# Supplementary material for: Baseline IL-6 is a biomarker for unfavorable tuberculosis treatment outcomes: a multi-site discovery and validation study
Source: Eur Respir J. Author manuscript; Available in PMC 2022 Jun 22. (PMC7612881; doi:10.1183/13993003.00905-2021)
Supplement: Supplementary Appendix [file EMS145909-supplement-Supplementary_Appendix.pdf]

Remove Watermark Now

Supplementary Appendix 1

Study population

Discovery cohort: The discovery cohort was designed for an exploratory analysis and its sample size was determined by project feasibility. Using a random number generator, we selected a sub-cohort of adults ($\geq$ 18 years) with newly diagnosed drug-sensitive pulmonary tuberculosis at the time of treatment initiation from the CTRIUMPH study at the Byramjee-Jeejeebhoy Government Medical College (BJGMC) in Pune(1). Cases were diagnosed by the presence of acid-fast bacilli (AFB) on smear microscopy, *Mycobacterium tuberculosis* DNA on Xpert MTB/RIF assay, *M tuberculosis* growth on culture, or based on clinical judgement in the absence of microbiological evidence of tuberculosis. HIV status was ascertained by ELISA (Genetic Systems HIV-1/HIV-2 PLUS O EIA, BioRad, USA). Diabetes was classified as having a prior physician diagnosis or a glycated hemoglobin (HbA1c) concentration $\geq$ 6.5% at enrollment. Chest X-ray (CXR) images were read by two independent reviewers and scored using a previously validated system which explicitly accounts for cavitation(2). Study participants received standard multi-drug tuberculosis treatment according to the Indian National Tuberculosis Elimination Program (NTEP) guidelines and, were followed for 24 months after treatment initiation to ascertain treatment outcomes of failure, recurrence or death. Plasma samples collected at treatment initiation, 2 months and 6 months underwent cytokine testing, in duplicates, using multiplex ELISA on Luminex assay (Bio-Rad, USA) at the National Institutes of Health (NIH) – National Institute for Research in Tuberculosis (NIRT) – International Center for Excellence in Research (ICER) laboratory in Chennai. Cytokines analyzed were selected a-priori for their role in the

pdfelement

Remove Watermark Now

host inflammatory response to *M tuberculosis* (interferon gamma [INF-γ], tumor necrosis factor alpha [TNF-α], interleukin [IL]-1β, IL-4, IL-6, IL-10, IP-10, IL-12, IL-13 and IL-17)(3), lung pathology (matrix metalloproteinases [MMP]-1, MMP-3, MMP-7, tissue inhibitor of metalloprotease [TIMP]-1, TIMP-2, TIMP-3, TIMP-4)(4), and fibrous remodeling (transforming growth factor beta [TGFβ]-1, TGFβ-2, TGFβ-3)(5).

Internal validation cohort: We nested a case-control study within the CTRIUMPH cohort to validate statistically significant results from the discovery analysis. Adults with culture confirmed drug-sensitive pulmonary tuberculosis were eligible for selection; those already included in the discovery analysis were excluded from internal validation. Cases comprised of pulmonary tuberculosis patients who failed treatment, defined as confirmation of *M tuberculosis* by culture during the last two months of treatment. Controls comprised of tuberculosis patients with two consecutive cultures negative for *M tuberculosis* during the last two months of treatment. Controls were matched to cases on age and sex in a 1:1 ratio. Socio-demographic, clinical and laboratory data were collected using standardized questionnaires and protocols as described in the discovery cohort. Study participants received standard multi-drug tuberculosis treatment according to NTEP guidelines. IL-6, IL-13 and IFN-γ measured at treatment initiation were statistically significantly associated with treatment failure in the discovery analysis and were therefore selected for internal validation. Plasma samples collected at treatment initiation underwent cytokine testing, in duplicates, using multiplex ELISA by Luminex assay (Bio-Rad, USA) at the BJGMC laboratory in Pune.

Indian external validation cohort: We nested a case-control study among adults with drug-sensitive pulmonary tuberculosis enrolled in the EDOTS cohort in Chennai(6).

pdfelement

Remove Watermark Now

Pulmonary tuberculosis was diagnosed by the presence of AFB on smear microscopy or culture confirmation of *M tuberculosis*; those with HIV coinfection were excluded. Participants received standard multi-drug tuberculosis treatment according to NTEP guidelines and were followed for 18 months after treatment initiation to ascertain outcomes. Cases comprised of participants with an unfavorable treatment outcome of failure, recurrence or death. Controls included participants with recurrence free cure during 18 months of follow-up. Controls were matched to cases on age, sex and BMI in a 2:1 ratio, with replacement. For participants reporting a prior history of diabetes, classification was confirmed by medical treatment history and HbA1c ≥ 6.5%. For participants with no known history of diabetes, classification was made by 75-gram oral glucose tolerance test as having a two-hour post-challenge blood glucose level ≥ 200mg/dL. CXR images were read by two independent reviewers and scored using a previously validated system which explicitly accounts for cavitation(2). IL-6 measured at treatment initiation was statistically significantly associated with treatment failure during internal validation and was therefore selected for independent external validation. Plasma samples collected at treatment initiation underwent IL-6 testing by Luminex assay (R&D Systems, USA) at the NIH-NIRT-ICER laboratory in Chennai.

South African external validation cohort: We nested a case-control study among adults with Xpert MTB/RIF-confirmed rifampin-susceptible pulmonary tuberculosis who were enrolled as part of a prospective cohort study in Khayelitsha, South Africa(7, 8). Participants received standard multi-drug tuberculosis treatment according to the South African National Tuberculosis Control Program guidelines and were followed for 22 months after treatment initiation. Cases comprised of participants with an unfavorable

pdfelement

Remove Watermark Now

treatment outcome of failure, recurrence or death. Controls included participants with recurrence free cure during 18 months of follow-up. HIV testing (Abbott Architect HIV Ag/Ab Combo test) and CD4+ T lymphocyte quantification were performed at enrollment. Diabetes was classified by a prior physician diagnosis or a HbA1c $\geq$ 6.5%. Plasma samples collected at tuberculosis treatment initiation underwent IL-6 testing by Luminex assay (MilliporeSigma, USA) at the Wellcome Centre for Infectious Disease Research, South Africa.

References

1. Gupte A, Padmapriyadarsini C, Mave V, Kadam D, Suryavanshi N, Shivakumar SV, et al. Cohort for Tuberculosis Research by the Indo-US Medical Partnership (CTRIUMPH): protocol for a multicentric prospective observational study. BMJ open. 2016;6(2):e010542.

2. Ralph AP, Ardian M, Wiguna A, Maguire GP, Becker NG, Drogumuller G, et al. A simple, valid, numerical score for grading chest x-ray severity in adult smear-positive pulmonary tuberculosis. Thorax. 2010;65(10):863-9.

3. O'Garra A, Redford PS, McNab FW, Bloom CI, Wilkinson RJ, Berry MP. The immune response in tuberculosis. Annu Rev Immunol. 2013;31:475-527.

4. Ong CW, Elkington PT, Friedland JS. Tuberculosis, pulmonary cavitation, and matrix metalloproteinases. American journal of respiratory and critical care medicine. 2014;190(1):9-18.

5. Tatler AL, Jenkins G. TGF-beta activation and lung fibrosis. Proc Am Thorac Soc. 2012;9(3):130-6.

pdfelement

Remove Watermark Now

6. Kornfeld H, West K, Kane K, Kumpatla S, Zacharias RR, Martinez-Balzano C, et al. High Prevalence and Heterogeneity of Diabetes in Patients With TB in South India: A Report from the Effects of Diabetes on Tuberculosis Severity (EDOTS) Study. Chest. 2016;149(6):1501-8.

7. Rockwood N, Costa DL, Amaral EP, Du Bruyn E, Kubler A, Gil-Santana L, et al. Mycobacterium tuberculosis Induction of Heme Oxygenase-1 Expression Is Dependent on Oxidative Stress and Reflects Treatment Outcomes. Front Immunol. 2017;8:542.

8. Rockwood N, Sirgel F, Streicher E, Warren R, Meintjes G, Wilkinson RJ. Low Frequency of Acquired Isoniazid and Rifampicin Resistance in Rifampicin-Susceptible Pulmonary Tuberculosis in a Setting of High HIV-1 Infection and Tuberculosis Coprevalence. The Journal of infectious diseases. 2017;216(6):632-40.

pdfelement

Remove Watermark Now

Supplementary Appendix 2

Supplementary table 1. Study schema for discovery, internal and external validation analyses.

	**Discovery**	**Internal validation**	**External validation 1**	**External validation 2**	**Pooled validation**
**Setting**	Pune, India	Pune, India	Chennai, India	Khayelitsha, South Africa	India and South Africa
**Design**	Prospective cohort study	Case-control analysis	Case-control analysis	Case-control analysis	Case-control analysis
**Sample size**	30 adults with drug-sensitive pulmonary tuberculosis	40 adults with drug-sensitive pulmonary tuberculosis	194 adults with drug-sensitive pulmonary tuberculosis	129 adults with rifampicin sensitive pulmonary tuberculosis	363 adults with drug-sensitive pulmonary tuberculosis
**Treatment outcomes**	4 with failure	20 with failure	18 with failure, 35 with recurrence and 19 deaths	9 with failure, 4 with recurrence and 5 deaths	47 with failure, 39 with recurrence and 24 deaths
**HIV coinfection, n (%)**	2 (7%)	3 (8%)	0	76 (59%)	79 (22%)
**Diabetes comorbidity, n (%)**	7 (23%)	3 (8%)	115 (59%)	10 (8%)	128 (35%)
**Laboratory**	NIH-NIRT-ICER, Chennai	BJGMC, Pune	NIH-NIRT-ICER, Chennai	Wellcome Center, Cape Town	BJGMC, NIH-NIRT-ICER and Wellcome Center

pdfelement

Remove Watermark Now

Supplementary table 2. Baseline characteristics and treatment outcomes of participants in the full cohort and those selected in the random sub-cohort for discovery analysis.

**Characteristics**	**Full cohort (n=445)**	**Sub-cohort (n=30)**	**p-value**
Age in years, median (IQR)	38 (27-49)	35 (28-51)	0.95
Male sex, n (%)	286 (64)	21 (70)	0.69
BMI in kg/m^2, median (IQR)	17.7 (15.8-20.4)	18.0 (15.8-19.9)	0.97
Ever-smokers, n (%)	144 (32)	8 (27)	0.68
HIV coinfection, n (%)	33 (8)	2 (7)	0.99
Diabetes, n (%)	99 (22)	7 (23)	0.82
Pre-treatment illness duration in days, median (IQR)	45 (30-90)	60 (20-90)	0.59
Percent of lung fields affected on CXR, median (IQR)	60 (25-80)	50 (24-68)	0.30
Cavitation on CXR, n (%)	167 (45)	10 (40)	0.68
Culture confirmed TB, n (%)	368 (84)	27 (90)	0.45
Smear positive TB, n (%)	305 (69)	20 (67)	0.84
Treatment outcomes, n (%)			
Failure	45 (10)	4 (13)	0.99
Recurrence	22 (5)	0	-
Death	20 (4)	0	-

TB – tuberculosis, IQR – interquartile range, BMI – body mass index, CXR – chest radiograph, n – sample size.

pdfelement

Remove Watermark Now

Supplementary table 3. Baseline characteristics of culture confirmed tuberculosis patients who failed treatment (cases) and those who were cured (controls) in the internal validation cohort.

Characteristics	Cases (n=20)	Controls (n=20)	p-value
Age in years, median (IQR)	27 (20-32)	26 (20-32)	0.92
Male sex, n (%)	15 (75)	15 (75)	0.99
BMI in kg/m^2, median (IQR)	16.0 (14.2-17.7)	17.4 (15.8-19.1)	0.02
Ever-smokers, n (%)	6 (30)	5 (25)	0.99
Alcohol dependence, n (%)	9 (45)	7 (35)	0.74
HIV coinfection, n (%)	2 (10)	1 (5)	0.99
Diabetes, n (%)	1 (5)	2 (10)	0.99
Pre-treatment illness duration in days, median (IQR)	35 (28-68)	30 (15-45)	0.03
Percent of lung fields affected on CXR, median (IQR)	58 (38-78)	60 (23-73)	0.43
Cavitation on CXR, n (%)	8 (40)	8 (40)	0.99
Smear positive TB, n (%)	14 (70)	11 (55)	0.51

TB – tuberculosis, IQR – interquartile range, BMI – body mass index, CXR – chest radiograph, n – sample size.

pdfelement

Remove Watermark Now

Supplementary table 4. Baseline characteristics of tuberculosis patients who experienced an unfavorable treatment outcome (cases) and those who were cured (controls) in the Indian external validation cohort.

**Characteristics**	**Cases (n=72)**	**Controls (n=122)**	**p-value**
Age in years, median (IQR)	45 (39-52)	45 (36-50)	0.40
Male sex, n (%)	64 (89)	104 (85)	0.52
BMI in kg/m^2, median (IQR)	16.8 (15.2-18.9)	17.2 (15.4-19.2)	0.58
Ever-smokers, n (%)	41 (57)	50 (41)	0.03
Diabetes, n (%)	42 (58)	73 (59)	0.88
Pre-treatment illness duration in days, median (IQR)	4 (4-8)	5 (4-8)	0.56
Percent of lung fields affected on CXR, median (IQR)	26 (15-40)	24 (14-40)	0.36
Cavitation on CXR, n (%)	18 (31)	30 (32)	0.99
Smear positive TB, n (%)	61 (91)	94 (90)	0.80
Culture confirmed TB, n (%)	64 (96)	104 (98)	0.40

TB – tuberculosis, IQR – interquartile range, BMI – body mass index, CXR – chest radiograph, n – sample size.

pdfelement

Remove Watermark Now

Supplementary table 5. Baseline characteristics of TB patients who experienced an unfavorable treatment outcome and those who were cured in the South African external validation cohort.

**Characteristics**	**Unfavorable outcome (n=18)**	**Cure (n=111)**	**p-value**
Age in years, median (IQR)	36 (30-48)	35 (30-43)	0.78
Male sex, n (%)	11 (61)	63 (57)	0.80
BMI in kg/m^2, median (IQR)	19.9 (19.0-23.0)	21.0 (19.1-23.3)	0.15
Ever-smokers, n (%)	8 (44)	52 (47)	0.99
Cavitation on CXR, n (%)	7 (39)	60 (54)	0.31
HIV coinfection, n (%)	13 (72)	63 (57)	0.30
ART receipt, n (%)	7 (39)	22 (20)	0.12
CD4 count, median (IQR)	178 (61-594)	194 (69-364)	0.98
Smear positive TB, n (%)	9 (50)	82 (74)	0.05

TB – tuberculosis, IQR – interquartile range, BMI – body mass index, CXR – chest radiograph, n – sample size, ART – antiretroviral therapy.

pdfelement

Remove Watermark Now

Supplementary table 6. Discriminatory ability of baseline IL-6 added to a risk-prediction model for unfavorable tuberculosis treatment outcomes.

**Baseline variables in the prediction model**	**AUC (95%CI) excluding baseline IL-6**	**AUC (95%CI) including baseline IL-6**	**p-value for gain in AUC by baseline IL-6**
Cavitation on CXR	0.52 (0.46-0.57)	0.68 (0.62-0.74)	<0.001
Smear grade $\geq$ 2	0.50 (0.45-0.55)	0.65 (0.59-0.71)	<0.001
BMI $\leq$ 18.5 kg/m^2	0.60 (0.51-0.70)	0.72 (0.62-0.82)	0.002
Cavitation and smear grade $\geq$ 2 and BMI $\leq$ 18.5 kg/m^2	0.66 (0.56-0.77)	0.76 (0.67-0.85)	0.02

AUC – area under the curve calculated by the C-statistic, CI – confidence interval, BMI – body mass index.

pdfelement
